# Arachidonic Acid Regulation of Intracellular Signaling Pathways and Target Gene Expression in Bovine Ovarian Granulosa Cells

**DOI:** 10.3390/ani9060374

**Published:** 2019-06-19

**Authors:** Nina Zhang, Liqiang Wang, Guoya Luo, Xiaorong Tang, Lizhu Ma, Yuxin Zheng, Shujie Liu, Christopher A. Price, Zhongliang Jiang

**Affiliations:** 1 College of Animal Science and Technology, Northwest A & F University, Yangling, Xianyang 712100, Shaanxi, China; ninazhang255@gmail.com (N.Z.); jhy12@126.com (L.W.); gagaluo23@163.com (G.L.); xiaortang@126.com (X.T.); malizhu@nwafu.edu.cn (L.M.); 18309256612@163.com (Y.Z.); 2 State Key Laboratory of Plateau Ecology and Agriculture, Key Laboratory of Plateau Grazing Animal Nutrition and Feed Science of Qinghai Province, Qinghai Plateau Yak Research Center, Qinhai University, Xining 810016, Qinghai, China; 13519705806@126.com; 3 Centre de recherche en reproduction fertility, Faculté de médecine vétérinaire, Université de Montréal, St-Hyacinthe, QC J2S 7C6, Canada; christopher.price@umontreal.ca

**Keywords:** arachidonic acid, granulosa cells, cell survival, lipid droplet, steroid hormone

## Abstract

**Simple Summary:**

Arachidonic acid (AA) is one of the polyunsaturated fatty acids that presents in a very high proportion in the mammalian follicular fluid. However, the mechanism of its effects on bovine ovarian granulosa cells is not clear. In the present study, we found that arachidonic acid plays an important role in regulating cell proliferation, lipid accumulation and steroidogenesis of granulosa cells. In this sense, arachidonic acid can directly affect the functionality of granulosa cells and therefore follicular development and ovulation, which could provide useful information for future studies relating to increasing fecundity of bovine.

**Abstract:**

In the present study, AA was used to challenge bovine ovarian granulosa cells in vitro and the related parameters of cellular and molecular biology were measured. The results indicated that lower doses of AA increased survival of bovine granulosa cells whereas higher doses of AA suppressed survival. While lower doses of AA induced accumulation of lipid droplet in granulosa cells, the higher dose of AA inhibited lipid accumulation, and AA increased abundance of *FABP3*, *CD36* and *SLC27A1* mRNA. Higher doses of AA decreased the secretion of E2 and increased the secretion of P4 accompanied by down-regulation of the mRNA abundance of *CYP19A1*, *FSHR*, *HSD3B1* and *STAR* in granulosa cells. The signaling pathways employed by AA in the stimulation of genes expression included both ERK1/2 and Akt. Together, AA specifically affects physiological features, gene expression levels and steroid hormone secretion, and thus altering the functionality of granulosa cells of cattle.

## 1. Introduction

Polyunsaturated fatty acids (PUFAs) play important roles as reservoirs of energy, as structural components of membranes and they are the precursors of steroid hormones [1]. Metabolites of PUFAs are also biologically active molecules and participate in different signaling cascades [1,2]. In the ovary, PUFAs play a critical role in maintaining membrane biogenesis, oocyte maturation and fertility in mammals [2,3].

Several studies have reported the PUFA composition in follicle fluid of cattle [4], humans [5], and pigs [6], and the most abundant PUFAs were linoleic acid (18:2, LA), and arachidonic acid (20:4, AA) [7]. Previous studies have demonstrated that AA was involved in the trophic hormone induced steroidogenesis [8,9]. In vertebrates, it has been demonstrated that AA and its metabolites could induce meiosis resumption in oocytes of toad [10] and fish [2]. Exogenous AA uptake resulted in activation of the COX pathway during ovulation, fertilization and embryogenesis [11,12]. It seems that AA is an essential fatty acid for follicular development, oocyte growth and fertility [6].

AA is cleaved from phospholipids by phospholipase A2 (PLA2) phosphatidyl inositol specific phospholipase C and the fatty acylglycerol lipase. AA then could metabolize to eicosanoids via cyclooxygenase (COX), lipoxygenase (LOX), or P450-dependent cyclooxygenase pathways [13]. A study in human follicular fluid showed that arachidonic acid derivatives impact oocyte intracytoplasmic sperm injection [14]. AA and its derivatives serve as cell-signaling intermediate, responsible for modulating cAMP activation, Ca^2+^ influx, Ca^2+^/CaM, PKC, MAPK and PI3K/AKT which results in regulation of cellular growth, proliferation, and differentiation [15,16,17,18,19,20,21].

In bovine ovary, the concentration of AA in follicle fluid accounts for approximately 2.5% of the total fatty acids, whereas it is 1.2 % in plasma [4]. There are some reports showing the effects of follicular AA on cumulus granulosa cells at the clinical level in both human [22] and non-human mammalian [7]. In human, Moran’s study indicated that overweight and obese women undergoing IVF (in vitro fertilization) became pregnant had higher levels of polyunsaturated fatty acid (PUFA) intake [22]. In cattle, Lapa’s study indicated that lower levels of arachidonic acid and conjugated linoleic acid (CLA) improved embryo quality by morphological evaluation [7]. However, the exact function and mechanism of action of AA on granulosa cells is unclear. The hypothesis of the present study was that AA affects the function of granulosa cell in cow’s ovary at the molecular level. Therefore, during this work, we studied the impact of AA on cell survival and apoptosis, lipid absorption, steroid hormone secretion, gene expression and intracellular signaling pathways involved in the function of granulosa cells with a model of in vitro cell culture.

## 2. Materials and Methods

The experimental protocol was approved by the Animal Care and Use Committee of the College of Animal Science and Technology, Northwest A&F University, Yangling, P.R. China.

### 2.1. Chemicals and Reagents

Unless stated differently, all chemicals and reagents used in this study were purchased from Sigma Chemical Co. (St. Louis, MO, USA). AA was purchased from Sigma-Aldrich, Life Science Inc. (St. Louis, MO, USA) and dissolved in 99.9 % ethanol to make the stock solution of 0.2 M.

### 2.2. Cells Culture and Treatment

Granulosa cells were cultured as described previously by Jiang et al. [23] with a few modifications. Briefly, 4–6 mm healthy follicles in the ovaries were obtained from adult dairy cattle, irrespective of estrous cycle stage at a local slaughterhouse were pierced with a 23 gauge needle and follicular fluid pooled together, then filtered through a 150 mesh steel sieve, (Sigma-Aldrich, Shanghai, China), centrifuged in 1500 rpm for 5 min. The number of cells was assessed using the Trypan blue dye exclusion procedure. Granulosa cells were seeded in 24-well tissue culture plates at a density of 5 × 10^5^ viable cells in 1 mL of DMEM/F12 containing sodium bicarbonate (10 mM), sodium selenite (4 ng/mL), bovine serum albumin (BSA) (0.1%, W/V, Sigma-Aldrich), penicillin (100 U/mL), streptomycin (100 μg/mL), transferrin (2.5 μg/mL), nonessential amino acid mix (1.1 mmol/L), bovine insulin (10 ng/mL), androstenedione (10^−7^ M) and bovine FSH (10 ng/mL, BIONICHE Inc. Ontario, Canada). Cultures were maintained at 37 °C in 5% CO_2_ and 95% air for 4 days. Every 2 days, 70% fresh medium was used to replace the medium in each well and treatments were then applied on Day 3. On the third day of cell culture, the stock solution of AA was diluted in fresh medium to treat the cells with the final concentrations of 1, 10, 50, 100 and 200 μM for 1, 4, 8, 16, 24, 48 and 72 h, respectively. The vehicle group of granulosa cells was treated with 0.1% (V/V) ethanol corresponding to the ethanol percentage of the highest AA treatment group (200 μM).

### 2.3. Assessment of Cell Survival and Apoptosis

Cell proliferation assays were performed with a Cell Counting Kit-8 (EnoGene, Nanjing, China) according to the manufacturer’s guidance. Setting a blank control group without cells, a control group containing cells but no AA, and a treatment group containing both cells and AA in a 96-well plate simultaneously. Cells were plated in triplicate at about 3 × 10^4^ cells per well. When cells confluence reached to 70%, cells was tested with different concentration of AA for same time or same concentration of AA for various time. At the end of AA treatment, 10 μL CCK-8 solution was added to each well and mixed gently. The numbers of cells per well were measured by the absorbance (450 nm) of reduced WST-8 (2-(2-methoxy-4-nitrophenyl)-3-(4-nitrophenyl)-5-(2,4-isulfophen-yl)-2H tetrazolium, monosodium salt) at the indicated time points.

The apoptosis of granulosa cells was analyzed by flow cytometry with an Annexin V-FITC apoptotic detection kit according to the instruction of the kit. Briefly, after treatment with 0.1% (V/V) ethanol (control) or different concentrations of AA, granulosa cells were harvested by 0.25% trypsin and washed twice in PBS (without Ca^2+^ and Mg^2+^), cells were resuspended with 100 μL 1× Binding Buffer at 1–5 × 10^6^ /mL and then added 5 μL Annexin V-FITC and 5 μL propidium iodide (PI) incubating for 10 min at room temperature, protected from light. Finally, 400 μL 1× Binding buffer was added and mixed gently. The granulosa cells apoptosis rate was analyzed using FACS (Becton Dickinson, San Jose, CA, USA) following standard protocols.

### 2.4. Oil Red O Staining

Intracellular lipid droplets were stained with Oil Red O. Cells were treated with 0.1% (V/V) ethanol (control) and different concentrations of AA as described. After treatment, the medium was removed and the cells were gently washed twice with 100 μL PBS. Then, 100 μL of 4 % paraformaldehyde in PBS was added to each well for cell fixation. After incubated for 30 min at room temperature, the paraformaldehyde was discarded and the cells were washed twice with 100 μL PBS. Subsequently, Oil Red O working solution was added to wells, cells were incubated at room temperature for 15 min. Then, Oil Red O solution was removed and the cells were washed twice with 100 μL PBS. Finally, the staining was evaluated in a Nikon TMS-F inverted microscope. For each group, three images were taken and analyzed for lipid area using ImageJ.

### 2.5. Steroid Production

After treatment, the culture medium was collected and stored at −20 °C until progesterone (P4) and 17β-estradiol (E2) concentrations were determined using ELISA kits (DIAsource, Louvain-la-Neuve, Belgium) in accordance with the manufacturer’s guidelines. The inter- and intra-assay CVs averaged 15% and 10.5%, respectively. The data represent three independent cultures with each treatment conducted in triplicate.

### 2.6. RNA Extraction and Real-Time PCR

After treatment, the culture medium was removed and total RNA was extracted using TRIzol. Total RNA was quantified by nanodrop-2000 (Thermo, Waltham, MA, USA) at 260 nm absorbance. Complementary DNA (cDNA) was synthesized using 5× All-In-One RT MasterMix (AccuRT Genomic DNA Removal Kit, abm, Richmond, BC, Canada) from 1 μg of RNA. The 20μL reverse transcription system included 2 μL AccuRT reaction Mix (4×), x μL RNA, 6-× μL Nuclease-free H_2_O, 2 μL AccRT Reaction Stopper (5×), 4 μL 5× All-In-One RT Master Mix and 6 μL Nuclease-free H_2_O. Reaction procedure comprised with initial incubation at 25 °C for 10 min, and then another incubation at 42 °C for 15 min, inactivation of the reaction at 85 °C for 5 min, 4 °C for preservation.

Real-time quantitative RT-PCRs were performed using the real-time PCR instrument (Bio-Rad, Hercules, CA, USA). Amplification reactions were performed in a 20 μL reaction volume containing 1 μL cDNA, 10 μL EvaGreen 2× qPCR MasterMix (EvaGreen 2× qPCR MasterMix-No Dye, abm), 7.8 μL RNase-free H2O, and 0.6 μL each of forward and reverse gene-specific primers. Cycle amplification conditions comprised an initial denaturation step at 95 °C for 10 min followed by 40 cycles at 95 °C for 15 s and 60 °C for 60 s. Immediately after amplification, PCR products were analyzed by sequencing, dissociation curve analysis. Gene expression was normalized to the Histone(H2AFZ)-internal control. The relative gene expression of the gene was calculated by 2−ΔΔCt method. Specific primer sequences were synthesized in Tsingke Biology Corp. (Xian, China). The primer sequences are shown in Table 1.

### 2.7. Western Blotting

After challenge with AA, cells were washed with cold PBS (pH 7.5) twice and lysed in 100 μL/well cold RIPA buffer (with 10 μL protease inhibitor cocktail and 10μL PMSF). The homogenate was transferred into a 1.5 mL tube and centrifuged at 10000× *g* for 5 min at 4 °C. Then, the supernatants were collected and BCA protein assay (Solarbio, Shanghai, China) was used to determine the sample concentrations.

Total 15 μg proteins/sample were resolved on 10% polyacrylamide gel by SDS-PAGE and electrophoretically transferred onto PVDF membranes in a Bio-Rad wet Blot Transfer Cell apparatus (transfer buffer: 39 mM glycine, 48 mM Tris-base, 1% SDS, 20% methanol, pH 8.3). After transfer, the membranes were washed and blocked with TBST (150 mM NaCl, 2 mM KCl, 25 mM Tris, 0.05% Tween20, pH 7.4) containing 5% BSA for 2 h at room temperature. Membranes were incubated overnight with the primary antibodies (anti-Akt, 1:1000, #9272; anti-phospho-Akt, 1:2000, #4060; anti-Erk,1:1000, #4695; anti-phospho-Erk,1:2000, #4370; Cell Signaling Technology, Danvers, MA, USA) in TBST containing 5% BSA at 4 °C. The membranes were then washed three times in TBST and incubated for 2 h at room temperature with anti-rabbit HRP-conjugated IgG (1:4000, LK2003, Sungene Biotechnology, Tianjin, China) diluted in 5% BSA in TBST. After three washes with TBST, protein bands on membranes were revealed by chemiluminescence (ECL, Millipore, Burlington, MA, USA) and autoradiography. Semiquantitative analysis was performed with NIH Image J software.

### 2.8. Statistical Analysis

All the experiments were performed in three replicates. All statistical analyses were performed using GraphPad Prism 6 software (GraphPad software Inc., San Diego, CA, USA). Duncan’s multiple range test by one-way analysis of variance (ANOVA) procedure was used to compare the mean values when the F-value was significant (*p* < 0.05). Experimental data are presented as the means ± SEM and differences with *p* values of less than 0.05 were considered statistically significant.

## 3. Results

### 3.1. Effects of AA on Survival and Apoptosis of Granulosa Cells

**Figure 1:**
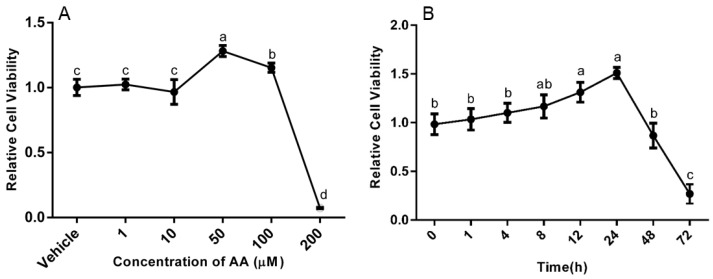
Effects of AA on the Viability in granulosa cells. Cells were challenged on Day 3 of culture with the doses given for 24 h in the left panel (**A**) or were challenged with 50 μM AA or not with AA for the times given in the right panel (**B**). Viable cells were assessed using cck-8 assay kit after treatments and data are means ± SEM of three independent replicates. For each panel, means without common letters are significantly different (*p* < 0.05).

We first surveyed the effects of AA on viability of granulosa cells from bovine follicles obtained at the slaughterhouse. On Day 3 of culture, cells were incubated with vehicle (0.1% ethanol) or with 1, 10, 50, 100 and 200 μM AA for 24 h, and the results are depicted in Figure 1A. The cell viability was significantly increased by the addition of 50 and 100 μM AA in comparison to the vehicle group (*p* < 0.05). There, however was no difference in cell viability between the groups treated with 1 or 10 μM AA and the vehicle group. Notably, addition of 200 μM AA significantly decreased cell viability compare with the vehicle treatment.

To determine the time-course of AA action on cell viability, cells were treated with 50 μM AA for 0, 1, 4, 8, 12, 24, 48 and 72 h. AA increased the viability of granulosa cells in a time-dependent manner from 0 to 24 h, but cell viability significantly decreased by 48 h and 72 h of addition of AA (Figure 1B).

**Figure 2:**
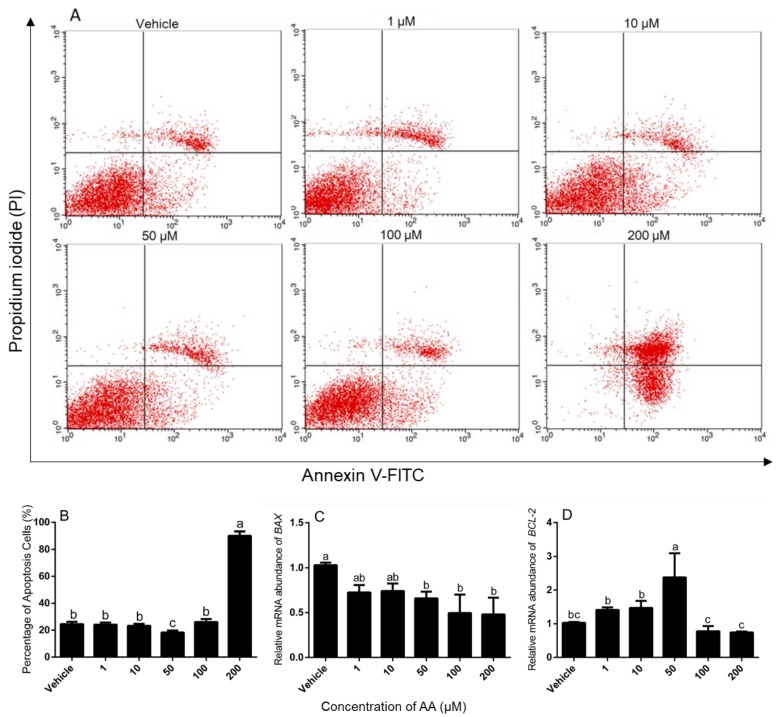
Effects of AA on the apoptosis in granulosa cells. Cells were challenged with increasing doses of AA for 24 h starting on Day 3 of culture. (**A**) The apoptotic cells were detected by flow cytometry. (**B**) Quantitative result of cells apoptosis as shown in A, ** *p* < 0.01. Abundance of mRNA encoding BAX (**C**) and BCL-2 (**D**), * *p* < 0.05. Data are means ± SEM of three independent replicates. For each treatment, means without common letters are significantly different (*p* < 0.05).

We then analyzed the effect of AA on apoptosis in granulosa cells. We found that granulosa cells had significantly higher number of apoptotic cells after treatment with 200 μM AA compared with vehicle controls, and 50 μM AA treatment had the lowest number of apoptosis cells, whereas none of the other doses altered the rate of apoptosis (Figure 2A,B). We also measured BAX/BCL-2 mRNA abundance and found that BAX mRNA abundance was decreased by treatment with 50, 100 and 200 μM AA compared with the vehicle (Figure 2C) and BCL-2 mRNA abundance was enhanced by 50 μM AA but not the other doses (Figure 2D).

### 3.2. Effects of AA on Fatty Acid Absorption by Granulosa Cells

**Figure 3:**
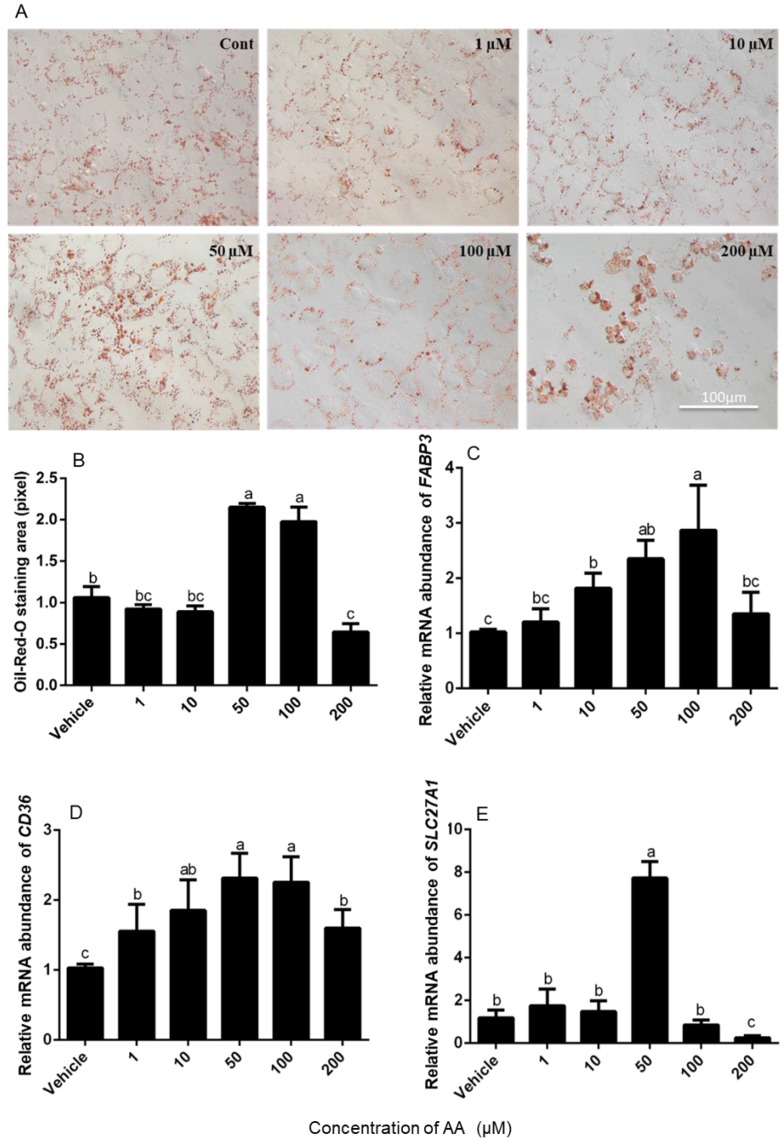
Effects of AA on the lipid droplet formation in granulosa cells. Cells were incubated with the doses given of AA for 24 h. Oil-Red-O staining was conducted as described in the methods section and red staining is indicative of lipid droplets in cultured granulosa cells with the doses given of AA for 24 h (**A**). Qualification of lipid droplets by ORO (Oil-Red-O) analysis in granulosa cells (**B**). FABP3 (**C**), CD36 (**D**) and SLC27A1 (**E**) mRNA abundance was determined in granulosa cells. Data are means ± SEM. of three independent replicates. For each treatment, means without common letters are significantly different (*p* < 0.05).

To determine whether AA altered absorption of fatty acids by granulosa cells, Oil-Red-O-staining of the cells was conducted after 24 h of treatment. The number of stained lipid droplets appeared abundant in cells that had been incubated with 50 and 100 μM AA compared with vehicle controls and 200 μM AA had less lipid droplets (Figure 3A). The quantitative visualization analysis further indicated that 50 and 100 μM AA stimulated lipid droplet accumulation in cells compared with vehicle, while 200 μM AA inhibited cells to accumulate lipid (Figure 3B).

We then measured the abundance of mRNA encoding *FABP3* (Figure 3C), *CD36* (Figure 3D) and *SLC27A1* (Figure 3E) in cultured granulosa cells, which are involved in the transport of fatty acids to appropriate intracellular compartments as cellular shuttles. The abundance of *FABP3* and *CD36* mRNA increased with increasing concentrations of AA up to 100 μM (*p* < 0.05; Figure 3C,D), whereas *SLC27A1* mRNA levels were increased by 50 μM AA but not by any other dose (Figure 3E). Interestingly, the highest dose of 200 μM AA decreased the abundance of mRNA encoding *SLC27A1* below the levels observed in vehicle controls (*p* < 0.05; Figure 3E).

### 3.3. Effects of AA on Steroidogenesis

**Figure 4:**
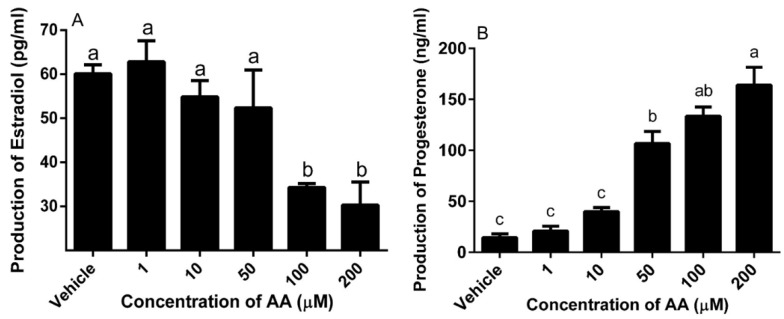
Effects of AA on the secretion of estradiol (**A**) and progesterone (**B**) in granulosa cells. Cells were challenged on Day 3 of culture with the given doses of AA for 24 h. Data are means ± SEM of three independent replicates. For each treatment, means without common letters are significantly different (*p* < 0.05).

To analyze the effect of AA on steroid production, culture medium of granulosa cells was collected to quantify steroid production after treatment with AA for 24 h. The higher concentrations of AA (100 and 200 μM) decreased the secretion of estradiol (*p* < 0.05) (Figure 4A), however significantly increased the secretion of progesterone (Figure 4B).

**Figure 5:**
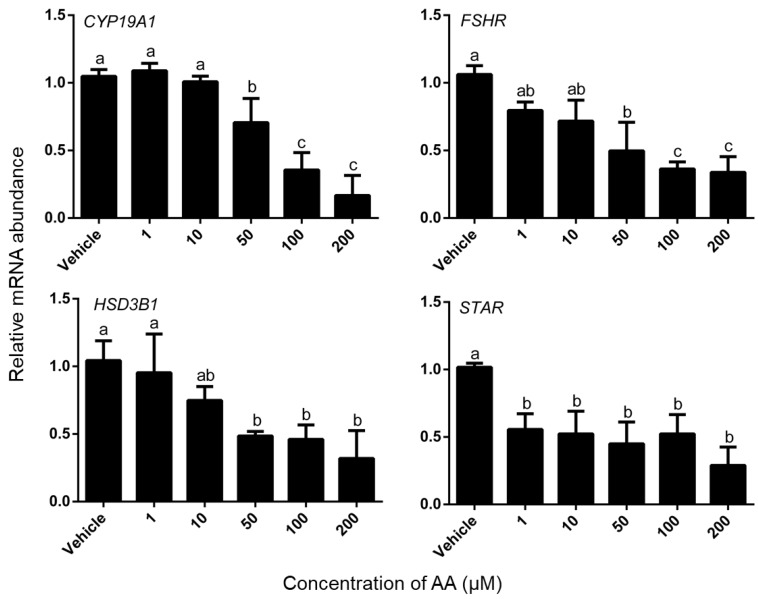
Effects of AA on the mRNA abundance of the related steroidogenic genes in granulosa cells. Granulosa cells were challenged on Day 3 of culture with the doses given of AA for 24 h. Data are means ± SEM. of three independent replicates. For each treatment, means without common letters are significantly different (*p* < 0.05).

The effect of AA on steroidogenic gene expression was measured in a separate experiment. Treatment with AA caused a dose-dependent decrease in abundance of *CYP19A1*, *FSHR HSD3B1* mRNA in granulosa cells and AA significantly decreased the expression level of *STAR* (Figure 5). For *CYP19A1*, *FSHR*, *HSD3B1*, the minimum effective dose was 50 μM, whereas 1 μM AA significantly decreased the abundance of *STAR* mRNA (*p* < 0.05).

### 3.4. Intracellular Pathways Activated by AA in Granulosa Cells

**Figure 6:**
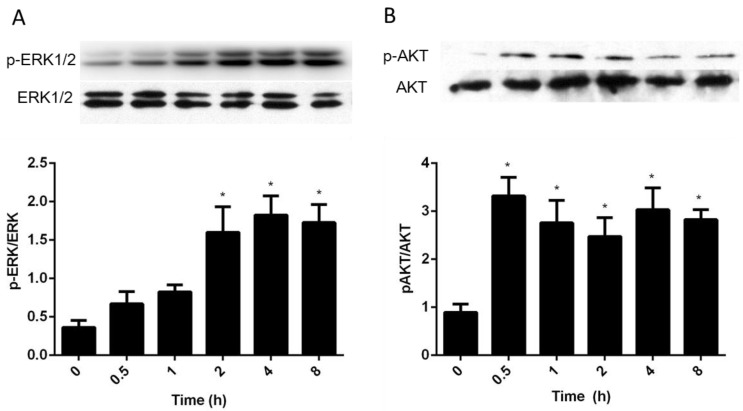
Effects of AA on the regulation of ERK1/2 and AKT phosphorylation in granulosa cells. Bovine granulosa cells were cultured in serum-free medium for three days and then treated with 50 μM AA for the times given. Phosphorylation of ERK1/2 (**A**) and AKT (**B**) in response to AA was measured by Western blot. Data are mean ± SEM of three independent replicates. * indicates significantly different from time 0 (*p* < 0.05).

To determine if AA activates the MAPK pathway in granulosa cells, 50 μM AA was used to treat the cells for 0, 0.5, 1, 2, 4 and 8 h, and Western blot was used to measure the abundance of intracellular phosphorylated ERK1/2. Addition of AA resulted in a significant increase in the levels of pERK1/2 between 2 and 8 h after treatment (Figure 6A, *p* < 0.05). We then determined the importance of PI3K/AKT signaling by Western blot studies. AA resulted in a significant and sustained increase in AKT phosphorylation in cultured bovine granulosa cells (Figure 6B).

## 4. Discussion

It has been suggested that dietary PUFAs have a pivotal role in modulating follicular development, steroidogenesis [24,25], and the follicle might have a local lipid metabolism that modulates ovulation and subsequent fertility [4]. AA is a major follicular PUFA in cattle [14,26]; however, little is known the specific role of AA on granulosa cells of cattle ovary. Therefore, this is the first study to report viability, gene expression, lipid droplet formation and steroidogenesis in bovine granulosa cells in response to AA treatment.

### 4.1. Effects of AA on Survival and Apoptosis of Granulosa Cells

In the present study, low doses of AA (50 μM) increased survival of bovine granulosa cells whereas higher doses (200 μM) suppressed survival. Our data is consistent with previous studies based on rat uterus stromal cells, in which AA (20 μM) significantly increased cell proliferation while a higher dose (100 μM) was cytotoxic [27]. Similarly, in colon cancer cell lines, it had been reported that 100 μM AA increased cell proliferation [28], but 200 μM AA, induced cell apoptosis [29]. Higher doses of AA (200 μΜ), equivalent to 10 times of physiological concentration of AA [26], induced large proportion apoptosis of cells, which may be correlated with oxidative stress: studies have found that arachidonic acid-induced cell death was directly implicated with mitochondrial damage caused by oxidative stress [30]. Toxic effects of high concentrations of fatty acids have also been described in oocytes [31] and in granulosa cells [32]. However, there is certain discrepancy with the results of the studies on human ovarian granulosa cells, which reported that AA had no effect on cell proliferation but protected cells from saturated fatty acid-induced cell apoptosis [33]. A possible explanation for this discrepancy might be the species or design of the protocols used. Our cell culture system was Fetal Bovine Serum (FBS) free, however, Mu adopted 10% FBS cell culture fluid. Another difference is the treatment time, the time for human ovarian granulosa cells was longer (3 days) than our experiment (1 day). The concentration of AA used in Mu’s experiment was lower (1–10 μM) than us. As far as we know, the concentration of AA in human follicular fluid is similar with cattle follicle.

The effect of high levels of AA (200 μM) on apoptosis may have acted through an altered BAX/BCL-2 ratio as treatment resulted in increased levels of proapoptotic factor *BAX* but not antiapoptotic factor *BCL-2* levels, although mRNA abundance does not necessarily reflect protein levels. In growing granulosa cells, BCL-2 plays important role in inhibiting mitochondria-mediated apoptosis, whereas the transcription of BAX is inhibited by estradiol. In atretic follicles, the expression pattern of these two genes in granulosa cells is revered [34].

### 4.2. Effects of AA on Lipid Droplet Accumulation in Granulosa Cells

Our study found that while 50 and 100 μM concentrations of AA induced accumulation lipid droplet in granulosa cells, the higher concentration of 200 μM inhibited lipid accumulation concomitant with the increased rate of apoptosis. As the increase in lipid accumulation was accompanied by an increase in the abundance of *FABP3*, *CD36* and *SLC27A1* mRNA, which are involved in the transport of fatty acids in different cell types [35,36], it is likely that AA acts by increasing the intracellular levels of these proteins. This is consistent with other studies in bovine granulosa cells, where oleic acid induced *CD36* and *SLC27A1* mRNA expression [37]. All the procedures of follicle development, from the recrutment of primordial follicle to growing pool to the initiation of follicle growth, to ovulation or leuteinization, require energy. In follicular foluid, fatty acids provide an improtant source of energy functioning through β oxidation [38]. Similarly, it has been observed that PUFAs can increase lipid droplet accumulation in a concentration-dependent manner in granulosa cells of cattle [37] and in Huh7 cells [39].

### 4.3. AA Down-Regulated mRNA Expression Level of Steroidogenesis-Related Gene

To understand the effect of AA on steroidogenesis of granulosa cells, we measured the secretion of E2 and P4 in cell culture medium. Our data showed that AA decreased the secretion of E2 at the concentration of 100 and 200 μΜ, and increased the secretion of P4 at the concentration of 50, 100 and 200 μΜ. One of the reasons for the decreased E2 concentration is the reduced steroidogenic enzyme mRNA level, such as the *CYP19A1*, *FSHR*, *HSD3B1* and *STAR*. Our results showed that AA down-regulated the expression of *CYP19A1*, *FSHR*, *HSD3B1* and *STAR* in cultured granulosa cells, which could explain the reason for the decrease of E2 secretion in granulosa cells. In similar studies, oleic acid has also decreased the abundance of *CYP19A1*, *FSHR*, *HSD3B1*, *STAR* mRNA and E2 production [37]. Our result is also consistent with previous studies based on granulosa cells, in which the increased secretion of P4 as the result of the increased apoptotic granulosa cells which continue to synthesize P4 until the breakdown of mitochondria [40].

In ovarian granulosa cells, CYP19A1 and FSHR are important factors that ensure the functional integrity of granulosa cells and the secretion of estrogen. As the final step of E2 synthesis, the expression of *CYP19A1* affected the production of E2 directly. The activation of FSHR by FSH leads to induce several target genes expression that are involved in processes of differentiation and proliferation in preovulatory follicles [41]. STAR, encoding steroidogenic acute regulatory protein, is involved in the initial step of steroidogenesis. This gene is one of the many genes that are activated by FSH. Subsequent steps are catalyzed by cholesterol side chain cleavage enzyme encoded by the 3 beta hydroxyl steroid dehydrogenase transcribed from HSD3B1 [42,43].

### 4.4. Intracellular Signaling Pathways Activated by AA

Here, we clarify that stimulation of survival and genes expression in granulosa cells treated by AA involves ERK1/2 and Akt phosphorylation. Our results shown that 50 μM of AA stimulated the proliferation of granulosa cells by activating both ERK1/2 and Akt signaling pathways that regulate target genes expression. In the mammalian oocyte, AA has the potential to stimulate cAMP/PKA by increased cPLA2 activity [44,45]. Maitra and colleagues concluded that PKA inhibition may have a potent role in MAPK (also known as extracellular signal-regulated kinases, ERK1/2) activation for initiation of oocyte maturation [46]. It is well known that both ERK1/2 and Akt as key proteins in MAPK and PI3K/Akt signaling pathways, which are involved in the regulation of cell proliferation and differentiation [47,48,49]. In follicle wall (theca and granulosa cells) samples, it was observed that there was more Akt and Erk 1/2 presenting in dominant follicles compared to the largest subordinate follicles [50].

## 5. Conclusions

In the present study, we demonstrated that AA regulates survival, gene expression, lipid formation, steroidogenesis and intracellular signaling pathways in cultured bovine granulosa cells. These data support the hypothesis that AA in the follicular fluid can directly affect the functionality of granulosa cells and therefore follicular development and ovulation.

## Figures and Tables

**Table 1:** Bovine specific sequences of primers used for real-time PCR.

Gene	(5′-3′) Forward Primer	(5′-3′) Reverse Primer	Accession No.
*H2AFZ*	GAGGAGCTGAACAAGCTGTTG	TTGTGGTGGCTCTCAGTCTTC	NM_174809
*FABP3*	CAGGCAGGTGGGCAATATGA	GCGTCACGATGGACTTGACTT	NM_174313
*CD36*	TGAGGCAGACACAACAAGAGT	ATCAGTGGTAACCAGTTGGAAGTC	NM_001278621
*SLC27A1*	CCAGGTCAGCCAGGGAACAA	CTCGCATCCTAGAGACCCTGAAG	NM_001033625
*FSHR*	AATTCATTTGTGCCAGCATCC	AGTTCGACCGCATCCCTG	NM_174061
*HSD3B1*	ACAATCTGACCGCATCGTCCT	CCACTTGCACCAGTGTCTTG	NM_174343
*CYP19A1*	GTGGACGTGTTGACCCTCAT	GGCACTTTCATCCAAGGGGA	NM_174305
*STAR*	GCCCAGAAACCTCAGCTCTTA	AGCTTTCCTGCTCCTAAGCAA	NM_174189
*BAX*	AACATGGAGCTGCAGAGGAT	CAGTTGAAGTTGCCGTCAGA	NM_173894
*BCL-2*	ATGACTTCTCTCGGCGCTAC	CTGAAGAGCTCCTCCACCAC	NM_001166486
